# Modeling progressive non-alcoholic fatty liver disease in the laboratory mouse

**DOI:** 10.1007/s00335-014-9521-3

**Published:** 2014-05-07

**Authors:** Jesse D. Riordan, Joseph H. Nadeau

**Affiliations:** Pacific Northwest Research Institute, Seattle, WA USA

## Abstract

Non-alcoholic fatty liver disease (NAFLD) is the most common liver disease in the world and its prevalence is rising. In the absence of disease progression, fatty liver poses minimal risk of detrimental health outcomes. However, advancement to non-alcoholic steatohepatitis (NASH) confers a markedly increased likelihood of developing severe liver pathologies, including fibrosis, cirrhosis, organ failure, and cancer. Although a substantial percentage of NAFLD patients develop NASH, the genetic and molecular mechanisms driving this progression are poorly understood, making it difficult to predict which patients will ultimately develop advanced liver disease. Deficiencies in mechanistic understanding preclude the identification of beneficial prognostic indicators and the development of effective therapies. Mouse models of progressive NAFLD serve as a complementary approach to the direct analysis of human patients. By providing an easily manipulated experimental system that can be rigorously controlled, they facilitate an improved understanding of disease development and progression. In this review, we discuss genetically- and chemically-induced models of NAFLD that progress to NASH, fibrosis, and liver cancer in the context of the major signaling pathways whose disruption has been implicated as a driving force for their development. Additionally, an overview of nutritional models of progressive NAFLD is provided.

## Introduction

Approximately one quarter of the global population meets the criteria for a diagnosis of non-alcoholic fatty liver disease (NAFLD), making it the most common liver disease in the world (Lazo and Clark 2008). Characterized by the presence of hepatic steatosis (fat accumulation in the cells of the liver) in the absence of excessive alcohol intake, NAFLD is often referred to as the hepatic manifestation of the metabolic syndrome (Marchesini et al. 2001). Accordingly, both obese and diabetic individuals are at particularly high risk, with estimates of prevalence in these populations as high as 70–90 % (Lazo and Clark 2008; Richard and Lingvay 2011; Starley et al. 2010). Additional risk factors include ethnicity, gender, dyslipidemia, and hypertension (Browning et al. 2004; Souza et al. 2012). A trend of steadily increasing incidence over the last three decades mirrors those of obesity and type 2 diabetes, demonstrating the growing significance of NAFLD as a major global health concern.

Though generally asymptomatic, NAFLD often progresses to more severe conditions, including non-alcoholic steatohepatitis (NASH), fibrosis, cirrhosis, and liver cancer. Indeed, its rising prevalence is a major factor contributing to the growing incidence of hepatocellular carcinoma (HCC) (Rahman et al. 2013; Siegel and Zhu 2009). The impact of NAFLD as an etiological factor for liver cancer is projected to expand in coming years as rates of obesity continue to rise, particularly in children. Since the likelihood of developing more severe liver pathologies is a function of the length of time an individual is affected, chronic exposure to a fatty liver environment from a young age confers an especially high risk of disease progression. Thus, the current childhood obesity epidemic is expected to promote an increased incidence of NAFLD-associated liver cancer in the near future.

Currently, reliable clinical biomarkers of progressive NAFLD are not available, and the molecular mechanisms driving disease advancement in humans remain inadequately characterized. As a result, cases are often not recognized until they reach a stage where attempts at curative treatment or disease stabilization are largely unsuccessful. While lifestyle changes such as diet modification and increased exercise are the primary recommendations for NAFLD patients, they are of limited benefit to patients with advanced liver disease. Gaining the ability to more effectively detect the progression of NAFLD to NASH, cirrhosis, and HCC is a major focus of ongoing research in hepatology. Another point of emphasis is the identification of molecular targets that can be exploited therapeutically to prevent or reverse disease.

Mouse models provide a powerful system with which to generate and test hypotheses about the molecular drivers of NAFLD progression to NASH, fibrosis, cirrhosis, and liver cancer. They allow a level of experimental control not possible with human studies, an essential factor in studying a complex, multifactorial condition such as NAFLD. The mouse genome has been characterized to a greater extent than any other animal model, and the resources available to researchers are unparalleled. The ease with which mouse colonies can be generated allows experiments to be conducted at sufficient scale to enable mechanistically and statistically meaningful conclusions. Additionally, the availability of inbred strains facilitates the design of precisely controlled experiments in which contributions of specific genetic elements to a phenotype of interest can be tested individually through minimization of the confounding effects of a genetically heterogeneous background. Another important advantage is the ability to control husbandry and environmental conditions, including diet and the microbiome, which allows standardization of important variables that can impact liver disease.

Several mouse models with phenotypic characteristics that closely resemble various aspects of progressive NAFLD in humans have been described. While no single model system completely recapitulates the entire spectrum of human NAFLD, they collectively provide a wealth of information on the molecular events that lead to the development of NASH, fibrosis, cirrhosis, and cancer in the context of a fatty liver environment. Furthermore, the ability to study individual phenotypic aspects of the disease through the use of distinct models can be highly advantageous, facilitating accurate interpretation of results by providing enhanced control over potentially confounding variables and allowing comparison across multiple experimental contexts.

In this review, we survey many existing mouse models of NAFLD, focusing on genetically, chemically, and nutritionally induced models that progress to inflammation, fibrosis, and in some cases liver cancer in the absence of further experimental insult. Distinct models are categorized based on major signaling pathways whose disruption has been identified as a driving force for disease development and progression. Table 1 provides a summary of the genetic models, their phenotypic characteristics, and their connection to human liver disease. We recognize that an abundance of additional mouse models have been described that develop progressive NAFLD through the combined effects of multiple etiological factors (e.g., transgenic or knockout strains that are sensitized to chemically- or nutritionally-induced NASH and HCC). Additionally, several examples have been reported of genetic modifications or drug treatments that confer protection in models that otherwise develop progressive NAFLD. An important role for the gut microbiota in influencing disease susceptibility and severity has also come to be appreciated (Aron-Wisnewsky et al. 2013; Dapito et al. 2012; Park et al. 2013; Yoshimoto et al. 2013). While these model systems have provided valuable insight into the molecular mechanisms of disease development and progression, they have been reviewed elsewhere (Anstee and Goldin 2006; Hebbard and George 2011; Nagarajan et al. 2012; Schattenberg and Galle 2010; Takahashi et al. 2012) and are not discussed here.

**Table 1:** Genetic models of progressive NAFLD in mice

Gene/Model	Modification	NASH	Fibrosis	Liver tumors	Observations in human liver disease
PI3K/AKT signaling					
*Pik3ca*	Liver-specific transgenic overexpression of activated mutant	No	No	Yes	Transgenic allele modeled after mutation detected in HCC
*Pten*	Liver-specific knockout	Yes	Yes	Yes	Decreased expression in NAFLD; Loss of function common in HCC
*Akt*	Liver overexpression via adenoviral or hydrodynamic injection	Yes	No	Yes	Increased expression in HCC associated with upregulation of lipogenic genes
JAK/STAT signaling					
*IL*-*6*	Constitutive knockout	Yes	No	No	^a^Increased expression in obesity and type 2 diabetes
*Il6st*	Constitutive knockout	Yes	Yes	No	^a^Activating mutations detected in liver cancer
*Jak2*	Liver-specific knockout	Yes	Yes	No	Sequence variants associated with metabolic syndrome risk
*Stat5*	Liver-specific knockout	No	No	Yes	Pathway activation associated with cirrhosis risk in NAFLD
PPAR signaling					
*Acox1*	Constitutive knockout	Yes	Yes	Yes	Sequence variants in PPAR associated with NAFLD
*Lgals3*	Constitutive knockout	Yes	Yes	Yes	Sequence variants in PPAR associated with NAFLD
NF-κB signaling					
*NEMO*	Liver-specific knockout	Yes	Yes	Yes	Decreased expression in HCC
*Atg5*	Systemic mosaic deletion	Yes	No	Yes	Autophagy is decreased in NAFLD and HCC
*Atg7*	Liver-specific knockout	Yes	No	Yes	Autophagy is decreased in NAFLD and HCC
*Becn1*	Systemic heterozygosity	Yes	No	Yes	Autophagy is decreased in NAFLD and HCC
Polygenic					
FLS strain	Selective breeding	Yes	Yes	Yes	
TSOD strain	Selective breeding	Yes	Yes	Yes	
SAM metabolism					
*Mat1a*	Constitutive knockout	Yes	No	Yes	Hypermethylation and decreased expression in NAFLD, cirrhosis, and HCC
*Bhmt*	Constitutive knockout	Yes	No	Yes	Decreased expression and loss-of-function mutation in HCC
*Gnmt*	Constitutive knockout	No	Yes	Yes	Hypermethylation and decreased expression in NAFLD, cirrhosis, and HCC

*NAFLD* non-alcoholic fatty liver disease, *NASH* non-alcoholic steatohepatitis, *HCC* hepatocellular carcinoma

^a^ Observation in humans is discordant with mouse model

## Characteristics of progressive NAFLD in humans

Histologically, NAFLD is defined as the presence of accumulated fat within the cytoplasm of at least 5 % of hepatocytes (Brunt and Tiniakos 2010). A lipid-specific stain, such as Oil Red O, can be used to visualize intracellular fat but is not always necessary due to the characteristic appearance of hepatic steatosis in samples prepared with standard histological techniques [e.g., hematoxylin and eosin (H&E) staining]. Macrovesicular steatosis is most commonly observed. Here, one or a few large fat droplets occupy the majority of a cell’s volume and distort the nucleus. Microvesicular steatosis is defined as the cytoplasmic accumulation of several small fat-containing vesicles that give the cell a foamy appearance without nuclear displacement. While this condition is more characteristic of acute liver damage (e.g., drug-induced toxicity) rather than chronic injury, it is observed in ~10 % of NAFLD patients (Tandra et al. 2011). Often co-occurring with macrovesicular steatosis, the presence of microvesicular steatosis has been linked to increased disease severity. For a diagnosis of NASH, hepatocyte injury and lobular inflammation must be present in addition to steatosis. Injury is usually detected by the presence of enlarged cells with diffuse cytoplasmic contents and a swollen appearance, a phenotype referred to as ballooning degeneration. This phenomenon is often caused by cytoskeletal disruptions, which can be identified by the loss of keratins 8 and 18, as detected by immunostaining (Lackner et al. 2008). Dense, eosinophilic structures made up of compacted cytoskeletal keratins called Mallory–Denk bodies are also common in cells undergoing ballooning degeneration.

In addition to histological evidence, liver injury may be indicated by the presence of elevated levels of liver enzymes alanine aminotransferase (ALT) and aspartate aminotransferase (AST) in the blood. However, liver enzymes are not always increased in NASH, and they may be increased in its absence. Therefore, histological analysis must be performed to establish a definitive diagnosis of NASH (Mofrad et al. 2003; Sorrentino et al. 2004). Inflammatory infiltration, which can be observed on an H&E stained section, is mostly lymphocytic. The presence of hepatic fibrosis is not required for a NASH diagnosis, but the two conditions frequently occur together. Characterized by the accumulation of excess extracellular matrix (ECM) and decreased cellularity, liver fibrosis results from a decline in normal matrix turnover and an increase in matrix deposition by activated hepatic stellate cells. It can be detected by histological procedures that distinctly stain collagen, the primary component of ECM. Commonly used techniques include Masson’s trichrome and Sirius Red staining. Additionally, the extent of stellate cell activation can be assessed by immunostaining for alpha-smooth muscle actin (α-SMA) (Feldstein et al. 2005). In some patients, fibrosis increases in severity to the point of cirrhosis, which is characterized by a loss of hepatic structural architecture, portal hypertension, and the formation of regenerative nodules.

An estimated 20 % of NAFLD cases advance to NASH (Attar and Van Thiel 2013), although the precise incidence of progression is difficult to determine because many cases are not recognized until advanced disease has already set in. This point is illustrated by the results of a study examining suspected NAFLD patients by biopsy, which is the gold standard for NASH diagnosis. Over half of those surveyed had definite NASH (Vuppalanchi et al. 2009). Approximately 15–25 % of NASH cases progress to liver cirrhosis, which is generally irreversible and represents a major risk factor for organ failure and HCC (Farrell and Larter 2006). In fact, the annual risk of HCC development in patients with cirrhotic NASH has been estimated at 2.6 % (Ascha et al. 2010).

## Mouse models of progressive NAFLD

### PI3K/AKT signaling

Dynamic regulation of the phosphatidylinositol 3-kinase/AKT thymoma viral proto-oncogene (PI3K/AKT) signaling pathway is central to a multitude of cellular processes. Of particular relevance to NAFLD development and progression is the pathway’s involvement in regulating glucose metabolism as well as cellular proliferation and survival. Mouse models of progressive NAFLD have been generated through genetic manipulation of specific factors involved at different points in the PI3K/AKT pathway, providing strong evidence for a causal link between perturbation of this signaling axis and disease development.

Near the top of the signaling cascade, the catalytic alpha subunit of PI3K (Pik3ca) relays activation signals from transmembrane receptor complexes by phosphorylating target lipid molecules to produce active secondary messengers. Transgenic mice with liver-specific overexpression of a mutant version of Pik3ca develop noticeably fatty livers by 4 weeks of age (Kudo et al. 2011). The transgene was designed to mimic a recurrent mutation detected in human HCC (Lee et al. 2005). In this model, severe hepatic steatosis accompanied by elevated serum ALT occurs by 6 months, and nearly all of the transgenic mice go on to develop liver tumors within 1 year. Consistent with these results, mice with liver-specific knockout of *Pik3ca* are protected from developing hepatic steatosis when fed a high fat diet (Chattopadhyay et al. 2011). In this study, deletion of an alternative PI3K catalytic subunit (*Pik3cb*) was found to have no significant effect on lipid accumulation within the liver, demonstrating a specific involvement of Pik3ca in this process. Manipulation of Pik3ca expression in either direction was associated with coordinate regulation of genes involved in lipogenesis (Chattopadhyay et al. 2011; Kudo et al. 2011), implicating fatty acid synthesis as a contributor to the observed phenotype. Induction of liver tumorigenesis by overexpression of mutant Pik3ca occurs in the absence of excessive weight gain, inflammation, or fibrosis (Kudo et al. 2011). Though unlike typical human progressive NAFLD in this regard, the model demonstrates that a steatotic liver can undergo neoplastic transformation without prior development of NASH or fibrosis. Models such as this may prove particularly useful for studying the subset of NAFLD-associated liver cancer in humans that arises in the absence of cirrhosis (Ertle et al. 2011).

Serving as an antagonist to the actions of PI3K, phosphatase and tensin homolog (Pten) dephosphorylates activated phosphatidylinositol secondary messengers to decrease output from the PI3K/AKT signaling pathway. Hepatic *Pten* deficiency promotes early-onset steatosis that increases in severity with age (Horie et al. 2004; Stiles et al. 2004). By 10 weeks, mice develop primarily microvesicular steatosis without other histological abnormalities and have normal levels of serum liver enzymes. In contrast to what is observed upon activation of Pik3ca, this model progresses to a NASH-like stage characterized by inflammation and fibrosis before eventually developing liver tumors. At 40 weeks, a mixed micro- and macrovesicular steatosis phenotype is present, along with Mallory–Denk bodies, ballooning degeneration, and elevated serum liver enzymes (Horie et al. 2004). Histologically, disease progression closely recapitulates the course of human NAFLD. Unlike the majority of human cases, however, mice exhibit decreased rather than increased extrahepatic adiposity (Stiles et al. 2004). The appearance of fatty liver is attributed primarily to activation of de novo lipogenesis, as suggested by significantly increased expression of genes involved in fatty acid synthesis (Horie et al. 2004) and directly demonstrated by quantitative experiments in which newly synthesized fatty acids were isotopically labeled in vivo (Stiles et al. 2004). In addition to functioning as an endoplasmic reticulum (ER) chaperone, heat shock protein 5 [Hspa5, also called glucose-regulated protein 78 and binding immunoglobulin protein (GRP78/BiP)] plays a regulatory role in the PI3K/AKT signaling pathway (Ni et al. 2011). Liver-specific co-deletion of *Pten* and *Hspa5* accelerates all aspects of disease progression (Chen et al. 2013b). Intriguingly, while NASH development in this model is associated with decreased Hspa5 expression, tumors arise from the proliferation of Hspa5-positive cells that have escaped Cre-mediated deletion. The authors suggest that loss of expression initially exacerbates steatosis and liver injury but that maintenance of expression provides cells with a selective advantage in the damaged liver environment, allowing them to avoid apoptosis and proliferate to form tumors. Pten is known to play a critical role as a tumor suppressor molecule. Targeted disruption of its function promotes tumorigenesis in a variety of tissues in mouse models, and it is one of the most frequently mutated genes in human cancer, including HCC (Hollander et al. 2011). Downregulation of PTEN has also been observed in hepatic steatosis in humans (Vinciguerra et al. 2008). Through liver-specific inactivation of *Pten* in mice, a direct role for this gene in regulating hepatic triglyceride dynamics was discovered.

Akt, also known as protein kinase B (PKB), is a serine–threonine protein kinase that acts as the primary effector molecule downstream of PI3K. Hepatic overexpression of activated Akt from an injected adenoviral expression construct resulted in significant fatty liver development in mice within 4 days (Ono et al. 2003). Though this study did not assess long-term effects on liver pathology, subsequent studies utilizing stably integrating transposon vectors delivered via hydrodynamic tail vein injection revealed that chronic overexpression of activated Akt causes mice to develop liver tumors in the context of hepatic steatosis. When delivered alone, activated Akt was sufficient to induce mixed micro- and macrovesicular steatosis and ballooning degeneration within 12 weeks that progressed to hepatocarcinogenesis within 28 weeks (Calvisi et al. 2011). Tumor formation could be accelerated through co-delivery of either β-catenin (Stauffer et al. 2011) or Nras (Ho et al. 2012). As has been observed in human HCC samples with aberrant activation of AKT (Calvisi et al. 2011), the development of severe hepatic steatosis was accompanied by enhanced expression of factors involved in lipogenesis in each of the hydrodynamic delivery models. Just one of these studies assessed inflammation and fibrosis, finding only the former to be present (Stauffer et al. 2011).

Whether through the direct overexpression of activated Pik3ca or Akt, or as a result of a loss of negative regulation by Pten, these murine models consistently demonstrate that hepatic activation of the PI3K/AKT signaling pathway promotes NAFLD development and progression to liver cancer.

### JAK/STAT signaling

The Janus kinase/signal transducer and activator of transcription (JAK/STAT) signaling cascade is another central pathway whose regulation is important for a variety of biological processes and whose disruption can cause progressive NAFLD. As a major mediator of cytokine signaling, functions of this pathway in the liver include response to injury and control of inflammation. Multiple mouse models of progressive NAFLD have been generated through targeted disruption of normal JAK/STAT signaling in the liver.

*Interleukin*-*6* (*IL*-*6*) is among the many cytokines that activate JAK/STAT signaling. Constitutive knockout of this gene causes mice to develop obesity and NASH (Matthews et al. 2010), which is driven in part by aberrant metabolic functions within hepatic mitochondria. Global deletion of *IL*-*6 signal transducer* (*Il6st*; also called gp130) produces a similar liver phenotype, characterized by steatosis, inflammation, and fibrosis (Betz et al. 1998). Although sensitized to inflammatory liver injury, mice with hepatocyte-specific *Il6st* knockout do not develop liver pathologies under basal conditions (Streetz et al. 2003), suggesting that loss of expression in other cell types contributes to the phenotype in constitutive knockout mice. Given that IL-6 levels are typically increased in NAFLD-associated conditions such as obesity and type 2 diabetes (Goyal et al. 2012; Roytblat et al. 2000) and that activating mutations in *IL6ST* have been recurrently detected in human liver cancer (Rebouissou et al. 2009), these results seem somewhat counterintuitive. Additionally, enhanced production of IL-6 has been demonstrated to functionally contribute to chemically-induced hepatocarcinogenesis in mice (Naugler et al. 2007). The precise role of this cytokine in metabolic liver disease is therefore controversial (Ouchi et al. 2011). Whether it promotes or protects from disease seems to vary depending on contextual factors, including cell type specific effects, acute versus chronic activation, and coordination of signals from additional cytokines.

Jak2 is a non-receptor protein tyrosine kinase that transduces signals from activated membrane receptor proteins to STAT molecules, which subsequently modulate transcription. Deletion of Jak2 function in a liver-specific manner causes mice to develop fatty liver by 4 weeks of age (Sos et al. 2011). Steatosis is initially macrovesicular, but becomes a mix of micro- and macrovesicular as it grows progressively worse with age. By 8 weeks the liver triglyceride content in conditional *Jak2* knockout mice is twenty times higher than that of control mice. NASH and fibrosis set in by 20 weeks, coincident with the appearance of elevated serum ALT and AST. Interestingly, these mice have a significantly lower extra-hepatic fat content than controls, suggesting a possible role for tissue redistribution of triglycerides in producing the steatotic phenotype. Consistent with this hypothesis, the Cd36 fatty acid transport protein was found to be significantly upregulated in liver. The authors went on to demonstrate that the effects of *Jak2* deletion on the liver could be prevented by eliminating global growth hormone (GH) signaling (a major activator of Jak2). They proposed a model wherein the absence of hepatic Jak2, by failing to propagate GH signals, causes a loss of feedback inhibition and leads to increased GH secretion by the pituitary. According to the model, this increased GH signal promotes lipolysis in adipose tissue, and the liberated fatty acids are transported into the liver for storage. Using the same model system, another group reported a similar steatotic phenotype, but without progression to inflammation and fibrosis (Shi et al. 2012). This study found hepatic *Jak2* deletion to be protective against high fat diet-induced liver inflammation. The authors attributed the disparate phenotypes to differences in genetic background. An association has recently been reported between polymorphisms in the sequence of *JAK2* and characteristics of the metabolic syndrome in humans (Penas-Steinhardt et al. 2011), supporting the relevance of these mouse models to human NAFLD.

STAT proteins function as dimeric transcription factors, translocating from the cytoplasm to the nucleus to regulate gene expression in response to JAK activation. Liver-specific deletion of *Stat5a* and *Stat5b* (*Stat5*) was found to cause NAFLD in mice (Cui et al. 2007). As is observed with hepatic *Jak2* deletion, mice have elevated levels of circulating GH, though a direct contribution to the phenotype was not investigated. Further characterization showed that mice develop liver tumors when aged to 17 months, possibly due to an impaired ability to respond to hepatic injury (Yu et al. 2012). Loss of liver *Stat5* expression also sensitizes mice to chemically-induced hepatic fibrosis (Hosui et al. 2009). Interestingly, human genome-wide association study (GWAS) data has identified the STAT5 signaling pathway as a potential modulator of cirrhosis risk in NAFLD patients (Chen et al. 2013a). Co-deletion of *Stat5* and glucocorticoid receptor [also called *nuclear receptor subfamily 3, group C, member 1* (*Nr3c1*)], a co-factor for a subset of Stat5 target genes, in hepatocytes produces liver tumors with decreased latency (Mueller et al. 2011). Mice develop combined micro- and macrovesicular steatosis associated with upregulated expression of lipogenic factors and increased serum ALT levels. Consistent with the model proposed by Sos and colleagues (Sos et al. 2011), elevated levels of circulating GH promote lipolysis in adipose tissue and hepatic uptake of fatty acids, which is associated with increased expression of *Cd36*. In each of the *Stat5* knockout models, expression of *Stat3* is increased. This effect has been mechanistically implicated in the development of liver disease upon deletion of *Stat5* (Hosui et al. 2009), demonstrating that the role of JAK/STAT signaling in progressive NAFLD involves a regulatory function that is more complex than simple gain or loss of function of the entire pathway.

### PPAR signaling

The peroxisome proliferator-activated receptor (PPAR) family of proteins consists of three nuclear hormone receptors that are important regulators of several NAFLD-related processes, including the synthesis, transport, and degradation of lipid molecules. Fatty acids and their metabolic derivatives act as ligands for PPARs, which upon binding heterodimerize with retinoid x receptors (RXRs) to form active transcriptional regulator complexes. With regard to fatty acid metabolism in the liver, PPAR-α and PPAR-β/δ mainly regulate catabolic functions, while PPAR-γ is primarily involved in anabolic functions (Yessoufou and Wahli 2010). Given their importance in hepatic lipid dynamics, it is not surprising that several variant *PPAR* sequences have been associated with NAFLD in humans (Tailleux et al. 2012). Additionally, genetically engineered mouse models have directly demonstrated a key role for aberrant signaling from this pathway in the development of progressive NAFLD.

Fatty acyl-CoA oxidase 1 (Acox1) is the rate-limiting enzyme that catalyzes the first step of peroxisomal fatty acid β-oxidation. Global deletion of this enzyme produces mice with microvesicular steatosis that develops within 2 months, progresses to steatohepatitis within 5 months, and is characterized by lymphocytic infiltration and hepatocyte death (Fan et al. 1996). Given the systemic nature of the model, the degree to which *Acox1* deletion in cell types other than hepatocytes contributes to the liver phenotype is unclear. Subsequent aging studies found that *Acox1*-null mice go on to develop liver fibrosis and HCC (Fan et al. 1998; Huang et al. 2011). The authors suggest that in the absence of Acox1, accumulation of fatty acid ligands causes hyperactivation of PPAR-α and that this increased output promotes hepatocarcinogenesis through excessive induction of oxidative and endoplasmic reticulum (ER) stress (Huang et al. 2011; Meyer et al. 2003). The necessity of PPAR-α for induction of the liver phenotype in *Acox1*-null mice is demonstrated by the finding that double knockout mice do not develop spontaneous steatohepatitis (Hashimoto et al. 1999). Further support for the conclusion that overactive PPAR-α signaling contributes to progressive NAFLD comes from the finding that synthetic xenobiotic activators of PPAR-α induce fatty liver and HCC with characteristics similar to various genetic models (Meyer et al. 2003; Reddy et al. 1980). Additionally, exposure to trichloroethylene (TCE) or perchloroethylene (PCE) causes steatohepatitis in mice, an effect attributed to the metabolic processing of these toxins to PPAR-α ligands (Wahlang et al. 2013).

Whereas PPAR-α is primarily involved in the breakdown of fatty acids, PPAR-γ signaling promotes their synthesis. This lipogenic activity of PPAR-γ has been implicated in hepatic steatosis development in mice through direct overexpression (Yu et al. 2003). Additionally, global knockout of galectin-3 (*Lgals3*), a member of the lectin family with a role in inflammation (Henderson and Sethi 2009), produces a fatty liver phenotype associated with PPAR-γ activation that progresses to fibrotic NASH and HCC (Nakanishi et al. 2008; Nomoto et al. 2006). As with the *Acox1*-null mice, this model’s systemic nature precludes the assignment of functional effects to specific cell types. Upregulation of PPAR-γ has also been mechanistically linked to steatohepatitis in models generated through genetic disruption of the PI3K/AKT (Horie et al. 2004; Kudo et al. 2011) and JAK/STAT (Mueller et al. 2011; Sos et al. 2011) signaling pathways, indicating the broad importance of this molecule in the pathogenesis of progressive NAFLD.

### NF-κB signaling

The nuclear factor-kappaB (NF-κB) pathway mediates several diverse biological processes. These include immune function, stress response, cell survival, and inflammatory signaling. In mammals, five NF-κB family member proteins interact to form dimeric complexes that act as transcription factors. These complexes translate signals from upstream molecules in the pathway [including interleukin-1beta (IL-1β), tumor necrosis factor alpha (TNF**-**α), and toll-like receptors (TLRs)] into a modified expression pattern for distinct sets of target genes. Progressive NAFLD can be induced in mice through genetic manipulation of independent regulatory molecules affecting NF-κB-mediated transcription, demonstrating involvement of this signaling pathway in disease development.

Carbon tetrachloride (CCl_4_) is a hepatotoxic agent that is commonly used to induce steatosis, inflammation, fibrosis, and liver cancer in mice. In addition to elevated serum ALT and AST levels, treated mice develop mixed micro- and macrovesicular steatosis, ballooning degeneration, Mallory–Denk bodies, and increased collagen deposition associated with hepatic stellate cell activation (Domitrovic et al. 2009). CCl_4_’s mechanism of action is complex and multifactorial, but is known to involve induction of oxidative stress and disruption of lipid metabolism (Weber et al. 2003). At the molecular level, activation of the NF-κB pathway via TNF**-**α signaling has been shown to be an important mediator of CCl_4_’s liver toxicity. Inhibition of either molecule’s function significantly decreases damage induced by this agent (Czaja et al. 1995; Wang et al. 1998).

Under basal conditions, NF-κB complexes are sequestered in the cytoplasm by inhibitor of κB (IκB) family proteins. Stimulatory input leads to their degradation, allowing NF-κB to enter the nucleus and alter transcription. This process results from phosphorylation of IκB by complexes of IκB kinase (IKK) and NF-κB essential modulator (NEMO) proteins, which leads to subsequent ubiquitination and proteasomal degradation. Liver-specific ablation of *NEMO* promotes highly penetrant HCC that is preceded by steatosis, NASH, and fibrosis (Luedde et al. 2007). In this model, the prevention of hepatic NF-κB activation causes many cells to undergo apoptosis, and compensatory proliferation of surviving cells generates a fatty liver that transitions to inflammatory fibrosis and HCC. Antioxidant treatment was found to eliminate this effect, demonstrating its dependence on oxidative stress. Loss of NEMO protein expression in HCC relative to adjacent tumor-free liver has been reported in humans (Aigelsreiter et al. 2012), further supporting a role as a suppressor of hepatocarcinogenesis.

Autophagy is an important cellular process that mediates degradation and recycling of intracellular components. Elimination of its function in the liver through the deletion of either *autophagy*-*related 5* (*Atg5*) or *autophagy*-*related* *7* (*Atg7*) causes mice to develop microvesicular steatosis with modest lymphocytic infiltration and multiple liver tumors (Singh et al. 2009; Takamura et al. 2011). Similarly, loss of a single *beclin 1* (*Becn1*) allele leads to NASH and HCC development (Mathew et al. 2009). These phenotypes arise, at least in part, as a result of impaired NF-κB activation in the autophagy-deficient liver. As with the *NEMO* knockout mice (Luedde et al. 2007), oxidative stress is thought to play a driving role in the liver pathogenesis of these models. This conclusion is consistent with data from human studies linking decreased autophagy to NAFLD and HCC (An et al. 2011; Fukuo et al. 2013; Mathew et al. 2009).

Activation of NF-κB signaling is an important component of NAFLD development in the context of hepatic ER stress (Gentile et al. 2011). Pharmacological ER stress induced by tunicamycin causes NASH in mice through a mechanism involving phosphorylation of IκB (Lee et al. 2012). Similarly, induction of ER stress by inhalation of airborne particulate matter derived from traffic pollution was found to promote NASH in mice via a mechanism involving TLR signaling and IκB phosphorylation (Laing et al. 2010; Zheng et al. 2013).

### Polygenic models

The targeted manipulation of individual genes to generate mouse models of progressive NAFLD is a powerful technique with several advantages, such as tight control over experimental variables, high reproducibility, and confident interpretation of results. A disadvantage of this approach is that it fails to recreate the complex etiology behind most cases of human disease. Rather than developing because of a dysfunctional inherited gene, human NAFLD is generally thought to result from the coordinate effects of several distinct molecules and pathways, along with contributions from environmental factors. In this regard, polygenic models more faithfully recapitulate the course of disease development. It is important to note that the genetic variants in these models are present ubiquitously and that atypical function in cell types other than hepatocytes may be involved in the liver phenotypes developed by these strains.

The fatty liver Shionogi (FLS) inbred mouse strain was derived from the Japanese ddN strain through repeated sibling mating of an outbred colony (Soga et al. 1999). These mice spontaneously develop microvesicular steatosis on a normal diet without becoming obese. NAFLD progresses to chronic NASH, which is associated with ballooning degeneration of hepatocytes, inflammatory infiltration, and fibrosis (Semba et al. 2013). FLS mice develop HCC with a latency of 16–24 months (Soga et al. 2003). Males are significantly more susceptible to liver disease than females, mirroring the trend in humans (Ferlay et al. 2010). Multiple molecular pathways related to fatty acid metabolism have been implicated as contributors to the liver phenotype in this strain. Chemical activation of PPAR-α decreases the severity of steatosis by activating a set of genes involved in fatty acid catabolism, including Acox1 (Harano et al. 2006). Along with the studies showing that hyperactivation of PPAR-α causes NASH (Fan et al. 1998; Huang et al. 2011; Meyer et al. 2003), this finding illustrates the dual role that PPAR-α activity may have as either a protective or promoting factor in progressive NAFLD depending on the context. Enhanced lipid transport function resulting from activation of microsomal triglyceride transfer protein (Mttp) also improves outcome in FLS mice by alleviating NASH symptoms (Shindo et al. 2010; Wang et al. 2014). Treatment with a non-steroidal anti-inflammatory drug (NSAID) protects from progression to HCC without affecting steatosis (Liu et al. 2006), revealing a key role for inflammatory signaling in driving hepatocarcinogenesis in this model.

Another polygenic mouse model of spontaneous progressive NAFLD is the Tsumura Suzuki obese diabetes (TSOD) strain. Similar to the FLS strain, these mice were generated through selective inbreeding of a Japanese outbred colony of ddY mice (Suzuki et al. 1999). Microvesicular steatosis develops in TSOD males by 4 months of age, and a mixed micro- and macrovesicular steatosis phenotype with ballooning degeneration and Mallory–Denk bodies is present at 6 months. By 1 year of age, livers display inflammatory infiltration, fibrosis, and tumor development (Nishida et al. 2013). Elevated hepatic levels of PPAR-γ were noted in this model, suggesting that excessive lipogenesis could be a factor contributing to fatty liver. It has also been suggested that impaired lipolysis plays a role in the phenotype of TSOD mice (Yogosawa et al. 2013).

### Nutritional models

One of the most commonly used methods to induce progressive NAFLD in laboratory mice is through dietary modification. Many variant diets have been used for this purpose. Key factors influencing the extent to which each diet promotes NAFLD, NASH, and HCC include the types and amounts of fat, carbohydrate, and cholesterol, as well as the presence or absence of critical nutrients, gender, strain background, and composition of the gut microbiome. Interactions between each of these factors ultimately determine the phenotypic outcome of feeding a modified diet. Given that dietary factors play a large role in human NAFLD, these models provide a relevant system in which to generate and test hypotheses about disease induction and progression.

Dietary formulations with the majority of calories derived from fats (typically 30–60 %) are referred to as high fat diets (HFDs). The increased fat content relative to a standard laboratory diet can trigger several symptoms of the metabolic syndrome, including obesity, hyperinsulinemia, hypercholesterolemia, and dyslipidemia (Buettner et al. 2007; Collins et al. 2004). HFDs directly provide an excess supply of fatty acids to the liver, where their storage generates conditions of steatosis. Continued exposure to an HFD can promote inflammation, fibrosis, and HCC (Hill-Baskin et al. 2009). Diets with increased cholesterol levels [e.g., the “Western diet” (TD.88137)] are also frequently used in studies of progressive NAFLD and can affect the liver phenotype (Matsuzawa et al. 2007).

In addition to its quantity, the source of dietary fat can significantly impact experimental outcomes. Because of their different compositions of polyunsaturated (PUFA), monounsaturated (MUFA), saturated (SFA), and *trans*-fatty acid (TFA), distinct sources of fat are subject to differential metabolic processing, which can lead to varying degrees of hepatic lipid accumulation (Ritze et al. 2013). Experimental evidence indicates that dietary SFAs and TFAs negatively impact liver health, while MUFAs and PUFAs are generally thought to be beneficial. A high dietary SFA content is associated with NASH in both mice and humans (Leamy et al. 2013; Toshimitsu et al. 2007). In mice, the mechanism of liver damage has been shown to involve hepatocyte apoptosis triggered by oxidative and ER stress (Leamy et al. 2013). High levels of dietary TFA also promote NASH in mice through a mechanism involving enhanced lipogenesis (Machado et al. 2010; Obara et al. 2010). Although a thorough assessment of the correlation between TFA consumption and NAFLD in humans has not been reported, associations with other characteristics of the metabolic syndrome have been made (Micha and Mozaffarian 2009). Dietary supplementation with MUFAs has been found to decrease liver fat content in diabetic patients (Bozzetto et al. 2012). The same effect has been observed in mouse models that develop NAFLD (Lee et al. 2011; Yang et al. 2011). In the context of a healthy liver, however, mice fed a diet high in MUFAs develop hepatic steatosis, although this effect is accompanied by a protective decrease in inflammatory signaling (Ferramosca et al. 2008; Guo et al. 2012). PUFAs, specifically the n-3 type (omega-3 fatty acids), have the most beneficial effects on liver health. Mechanistically, they negatively regulate lipogenesis and inflammatory signaling while promoting fatty acid breakdown (Di Minno et al. 2012). Depletion of PUFAs from the diet induces fatty liver in mice (Pachikian et al. 2011), and their supplementation can prevent or reverse steatosis (Alwayn et al. 2005; Sekiya et al. 2003). Clinical trials have also demonstrated success in decreasing hepatic fat content, serum liver enzymes, hepatocyte damage, and inflammation in NASH patients treated with a diet high in n-3 PUFAs (Capanni et al. 2006; Tanaka et al. 2008).

Carbohydrate content is another dietary factor that can significantly influence the course of disease development in progressive NAFLD. Ketogenic diets (KD) have a very low amount of total carbohydrates, and they are typically high in fat. Mice fed such a diet develop steatosis with faster kinetics than mice fed a “Western diet” with high levels of fat and simple carbohydrates (Garbow et al. 2011). KD-fed mice go on to develop NASH without becoming obese. Dietary carbohydrates can be broadly classified as either simple (sugars) or complex (starches), and each has distinct effects on metabolic syndrome-associated conditions (Aller et al. 2011). Simple carbohydrates are more easily digested and absorbed, as complex carbohydrates must be broken down into their constituent sugars before they can be processed further. Diets high in simple carbohydrates have been found to promote steatosis in mice more readily than matched diets with complex carbohydrates (Isken et al. 2010).

Of the major dietary sugars, fructose has been found to be a particularly strong driver of NAFLD and NASH (Basaranoglu et al. 2013; Nomura and Yamanouchi 2012). One reason for this effect is its efficient uptake and metabolic processing by the liver. Solute carrier family 2, facilitated glucose transporter member 5 (Slc2a5 or Glut5) is a membrane transport protein that specifically facilitates the uptake of dietary fructose from the intestinal lumen into the portal circulation. Along with family member Slc2a2/Glut2, which additionally transports glucose and galactose, Slc2a5 efficiently moves fructose from the bloodstream into hepatocytes in an insulin-independent manner. In the liver, fructose is rapidly metabolized by glycolytic and lipogenic pathways through a mechanism that bypasses a major rate-limiting step utilized in glucose metabolism. Mice on a high fructose diet exhibit increased hepatic expression of lipogenic proteins, elevated serum ALT, and NASH characterized by mixed micro- and macrovesicular steatosis after 16 weeks (Schultz et al. 2013). These phenotypes develop in the absence of obesity and, at least at this experimental time point, are not significantly increased in severity by the addition of a high fat content to the high fructose diet. Supplementation of other NAFLD models with a high fructose diet can significantly increase the severity of developed liver pathologies (Charlton et al. 2011; Dowman et al. 2014; Pickens et al. 2010; Tetri et al. 2008). Excessive fructose intake has also been linked to the occurrence and severity of NAFLD in humans (Abdelmalek et al. 2010; Ouyang et al. 2008; Zelber-Sagi et al. 2007). Given the high level of human fructose consumption, mouse models of progressive NAFLD incorporating this variable are likely to provide valuable mechanistic insight with high relevance to human disease.

Methionine, choline, and folate are essential nutrients, which means that they cannot be synthesized de novo by the body and must be included in the diet. Feeding mice a methionine- and choline-deficient (MCD) diet leads to aggressive, early-onset NASH and fibrosis that is mechanistically linked to oxidative stress (Gao et al. 2004; Rinella and Green 2004). Methionine deficiency alone can cause fibrotic NASH, while choline deficiency cannot, though it does significantly increase the extent of steatosis in the context of methionine deficiency (Caballero et al. 2010). Co-deficiency of dietary choline and folate results in NAFLD that variably progresses to NASH and fibrosis depending on strain background (Pogribny et al. 2013).

A potential unifying factor underlying these distinct dietary models is the mechanistic involvement of disruption in the metabolism of S-adenosylmethionine (SAM), which has many cellular functions important for liver health (Lu and Mato 2012). Methionine is the direct metabolic precursor to SAM, and its levels are replenished by metabolic processing of choline and folate. As such, deficiency of these nutrients causes SAM depletion. Restoration of hepatic SAM levels reduces the severity of liver disease induced by the MCD diet, indicating the importance of this molecule in the model system (Henkel et al. 2009). SAM serves as the primary methyl donor for a variety of biological substrates and can be metabolically processed to generate the critical antioxidant glutathione (GSH).

Several genetic models have been generated that demonstrate the importance of SAM regulation in maintaining liver health (Lu and Mato 2012). Constitutive knockout of *methionine adenosyltransferase 1 alpha* (*Mat1a*), which encodes the enzyme responsible for converting hepatic methionine to SAM, results in its depletion and promotes spontaneous NASH that progresses to HCC (Lu et al. 2001; Martinez-Chantar et al. 2002). Oxidative stress is high in this model due to a deficiency of GSH. The relevance of this model to human disease is demonstrated by studies identifying hypermethylation of the *MAT1A* promoter region and associated decreased expression in the livers of patients with NAFLD and HCC (Frau et al. 2012; Murphy et al. 2013). A similar liver phenotype develops in mice with constitutive knockout of *betaine*-*homocysteine S*-*methyltransferase* (*Bhmt*). Bhmt is an enzyme that generates methionine by transferring a methyl group from betaine (a choline derivative) to homocysteine, and hepatic SAM levels drop by over 40 % in its absence (Teng et al. 2011). Loss-of-function mutations in *BHMT* have been detected in human HCC, suggesting that its absence may contribute to hepatocarcinogenesis (Pellanda et al. 2012). Steatosis, fibrosis, and HCC develop spontaneously in mice deficient for *glycine N*-*methyltransferase* (*Gnmt*), which encodes an enzyme that catalyzes the transfer of a methyl group to glycine as a way to regulate cellular SAM levels and prevent aberrant methylation of other substrates (Martinez-Chantar et al. 2008). In its absence excessive levels of SAM accumulate in the liver, leading to excessive methylation of DNA and histones. Data from human studies similarly suggest that *GNMT* acts as a tumor suppressor in the liver. Its expression is frequently decreased in HCC in association with hypermethylation of the locus (Chen et al. 1998; Huidobro et al. 2013). Additionally, *MAT1A*, *BHMT*, and *GNMT* were found to be coordinately downregulated in human cirrhosis and HCC, along with other genes involved in SAM metabolism (Avila et al. 2000). As demonstrated by these mouse models and their connection to human liver disease, maintenance of normal SAM levels is critical; either a deficiency or an excess can cause liver pathologies including NASH, fibrosis, and HCC.

## Insights gained from mouse models of progressive NAFLD

Mouse models of progressive NAFLD have provided many valuable insights into the pathogenesis of this prevalent human disease, increasing our understanding of its environmental and molecular drivers. Such knowledge is critical for the development of improved diagnostic, prognostic, preventative, and therapeutic approaches. The generation of model systems through genetic targeting to manipulate specific signaling pathways has definitively demonstrated their importance in NAFLD development and progression, with comparison to human disease verifying their clinical relevance. As reviewed here, the PI3K/AKT, JAK/STAT, PPAR, and NF-κB pathways have emerged as central regulators of progressive NAFLD. However, crosstalk between these and additional signaling pathways is certain to have a role in the pathogenesis of this complex disease. Examples include activation of PPAR-γ and mitogen-activated protein kinase (MAPK) signaling in the liver of mice with genetic PI3K/AKT pathway activation or JAK/STAT pathway deficiency (Horie et al. 2004; Mueller et al. 2011; Sos et al. 2011) and the involvement of upregulated NF-κB and hedgehog (Hh) signaling pathways in MCD diet-induced NASH (Dela Pena et al. 2005; Syn et al. 2012). It is becoming increasingly evident that the ultimate determination of liver phenotype in NAFLD depends on the integration of several signals coming from distinct pathways and cell types.

Mouse models have also helped to elucidate key cellular processes underlying the development of progressive NAFLD, including oxidative and ER stress (Malhi and Kaufman 2011; Rolo et al. 2012). Activation of the ER stress response is associated with NAFLD caused by a wide variety of distinct molecular mechanisms, and it has been mechanistically linked to both the induction of hepatic steatosis and its progression to more severe liver damage (Zhang et al. 2014). The effects on steatosis are mediated primarily through enhanced lipogenesis, while advancement to NASH is driven by increased apoptosis and oxidative stress-associated inflammation within the liver. The shared contribution of processes such as oxidative and ER stress to progressive NAFLD serves to mechanistically unite seemingly disparate model systems. It also suggests that therapeutic approaches designed to minimize the damaging effects of these processes may be especially efficacious.

The importance of genetic background in determining the degree of susceptibility to genetically or environmentally induced progressive NAFLD is another key insight provided by mouse models. Several studies have demonstrated profound interstrain variability in hepatic phenotypes induced by liver damaging treatments (Hill-Baskin et al. 2009; Kahle et al. 2013; Montgomery et al. 2013; Pogribny et al. 2013; Shockley et al. 2009; Tryndyak et al. 2012; Yamazaki et al. 2008). Similar gene-environment interactions undoubtedly factor into the pathogenesis of human NAFLD. Indeed, genetic factors involved in a diverse array of cellular processes have been identified as contributors to human NAFLD susceptibility in recent years (Al-Serri et al. 2012; Carulli et al. 2009; Chalasani et al. 2010; Gawrieh et al. 2012; Musso et al. 2013; Romeo et al. 2008; Yoneda et al. 2008).

Knowledge gained through the use of mouse models of progressive NAFLD has improved our understanding of this disease in humans. Clarification of the major forces promoting disease development in a variety of genetic and environmental contexts has led to the identification of promising diagnostic markers and therapeutic targets. These and future discoveries made using mouse models will help to alleviate the health burden imposed by the rising incidence of progressive NAFLD worldwide.
